# The search for spontaneous edge currents in Sr_2_RuO_4_ mesa structures with controlled geometrical shapes

**DOI:** 10.1038/s41598-023-39590-9

**Published:** 2023-08-04

**Authors:** P. J. Curran, S. J. Bending, A. S. Gibbs, A. P. Mackenzie

**Affiliations:** 1 Department of Physics, University of Bath, Claverton Down, Bath, BA2 7AY UK; 2 School of Chemistry, University of St. Andrews, St. Andrews, KY16 9ST UK; 3 School of Physics and Astronomy, University of St. Andrews, St. Andrews, KY16 9SS UK; 4 Max-Planck Institute for Chemical Physics of Solids, 01187 Dresden, Germany

**Keywords:** Superconducting properties and materials, Imaging techniques

## Abstract

Scanning Hall microscopy has been used to search for spontaneous edge fields in geometrically shaped mesa structures etched into the *ab* surface of Sr_2_RuO_4_ single crystals in order to test recent theories of the direction of edge current flow as a function of facet orientation and band filling. We find no evidence for spontaneous edge fields in any of our mesa structures above our experimental noise floor of ± 25 mG. We do, however, observe pronounced vortex clustering at low fields and temperatures, consistent with the established semi-Meissner scenario whereby a long range attractive component to the vortex-vortex interaction arises due, for example, to the multiband nature of the superconductivity. We also see clear evidence for the formation of a square vortex lattice inside square mesa structures above 1.3 K. Our results are discussed in terms of recent relevant experimental results and theoretical predictions.

## Introduction

Soon after the first discovery of superconductivity in Sr_2_RuO_4_ in 1994^1,2^ it was identified as a strong potential candidate for unconventional spin-triplet superconductivity. Experimental evidence for this came from early NMR Knight-shift measurements under in-plane magnetic fields indicating that the shift remains unchanged as the temperature is lowered into the superconducting state^3^. In addition, muon spin rotation (µSR)^4^ and polar-Kerr measurements^5^ showed evidence for time reversal symmetry breaking (TRSB) identifying a two-component chiral p-wave order parameter $\hat{\user2{d}} = \Delta_{0} \left( {{\varvec{k}}_{{\varvec{x}}} \pm {\varvec{ik}}_{{\varvec{y}}} } \right)\hat{\user2{z}}$ as a likely candidate. However, this description appeared to be in conflict with experimental evidence from thermal conductivity^6^ and specific heat measurements^7^ suggesting a nodal gap structure, while uniaxial strain measurements did not reveal the split superconducting transition expected for a chiral p-wave state^8^. The original Knight shift measurements were recently revisited taking care to avoid sample heating due to high amplitude radio frequency pulses and have indeed shown a reduction in Knight shift below the critical temperature^9,10^. Together with subsequent ^17^O NMR Knight shift measurements on Sr_2_RuO_4_^11^ these appear to rule out all odd parity order parameter states, regardless of the $\hat{d}$ vector orientation. More recently ultrasound experiments by Ghosh et al.^12^ and Benhabib et al.^13^ have provided thermodynamic evidence that Sr_2_RuO_4_ exhibits a two-component order parameter. Taking either the observation of TRSB or gap nodes as key additional information, these authors variously propose time reversal breaking or time reversal invariant order parameters respectively. Clearly our understanding of superconductivity in this remarkable material is still far from complete and further experimental measurements are required to gain deeper insights into this problem.

**Figure 1:**
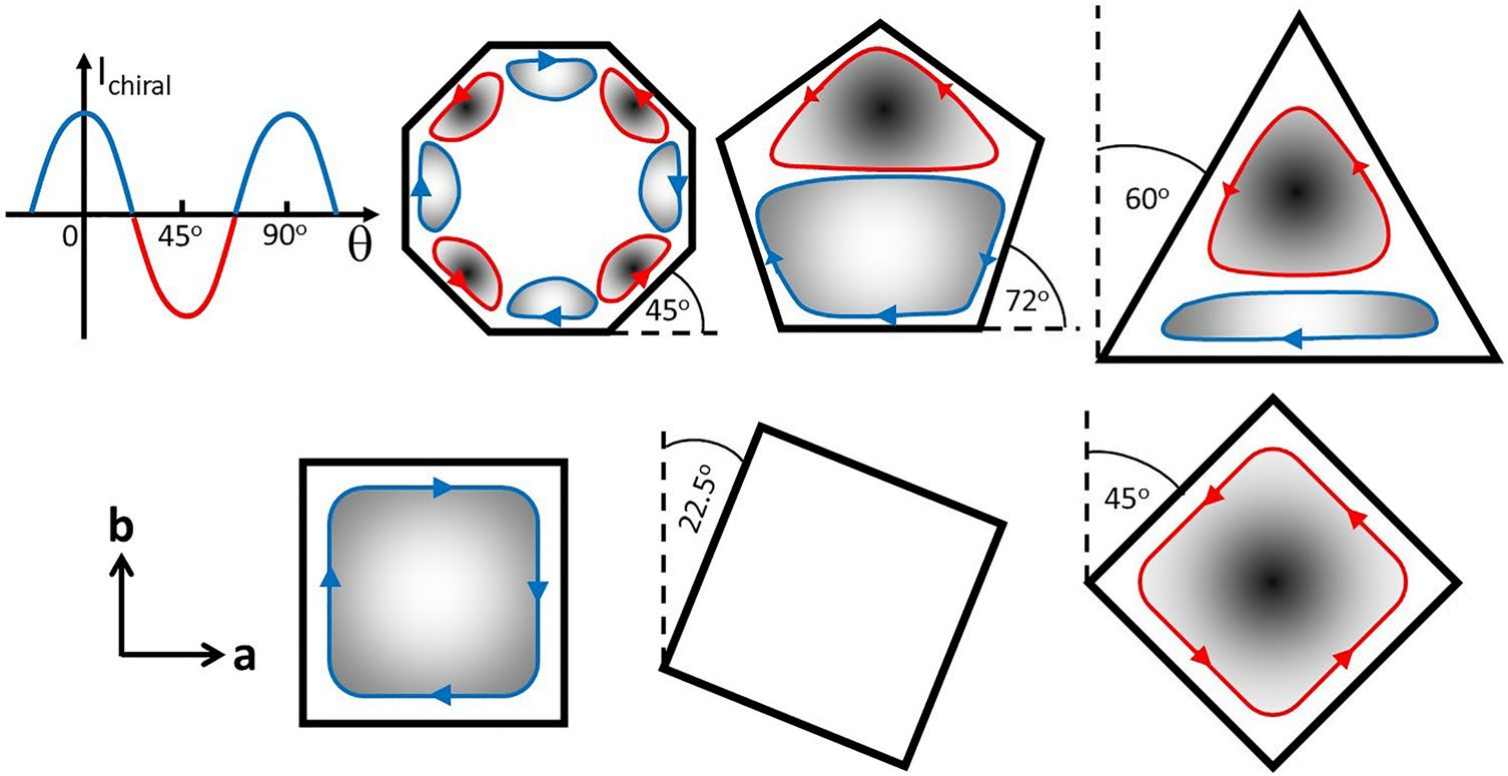
Model of edge currents as a function of facet angle, θ, and sketches of the expected edge fields for different mesa geometries and orientations.

If Sr_2_RuO_4_ did exhibit a chiral superconducting phase which breaks time-reversal symmetry it is predicted to host spontaneous currents at the sample surface or chiral domain walls. These surface currents are expected to produce local magnetic fields which should be detectable with low temperature scanning probe techniques, yet all reported experiments have so-far failed to resolve them^14–16^. To address this issue, in particular a null result for Sr_2_RuO_4_ single crystals with microscopic cylindrical pillars etched into their surface^17^, Bouhon et al.^18^ have made a detailed theoretical study of the geometry and band structure dependence of the edge states. Using a tight-binding model of a square lattice for the Sr_2_RuO_4_ γ bands, they solved the Bogolyubov-de Gennes equation self-consistently assuming a chiral p-wave superconducting state. Their results reveal that the edge state dispersion depends strongly on both the orientation of the surfaces and the band filling. At T = 0 and low band filling, currents at both the {1,0,0} (θ = 0°) and {1,1,0} (θ = 45°) crystal surfaces are predicted to flow in the same + k_//_ direction, while at high band filling currents at {1,1,0} reverse and propagate in the opposite sense to those at {1,0,0} surfaces. For Sr_2_RuO_4_ the band filling is expected to be rather large when the latter scenario applies, and the edge fields will be screened away from the surface/domain walls over a characteristic length of the London penetration depth. Assuming that the edge current density depends approximately sinusoidally on the facet angle, the consequence for a number of different sample geometries is sketched in Fig. 1. For the octagonal sample the direction of edge currents reverses at every adjacent facet leading to very weak edge fields that reverse sign periodically around the perimeter of the structure, while the regular pentagon or equilateral triangle would be expected to show weak dipole field distributions. From an experimental point of view the most interesting geometry is that of a square, θ = 0° and θ = 45° squares are expected to have edge currents propagating in opposite directions around the perimeter, while a θ = 22.5° square should almost have zero edge current density at the surface. Motivated by these results we report here a systematic search for edge currents around mesas with various geometrical shapes etched into the surface of a Sr_2_RuO_4_ single crystal with facets at well-defined angles with respect to the underlying crystallographic axes.

## Experimental methods

**Figure 2:**
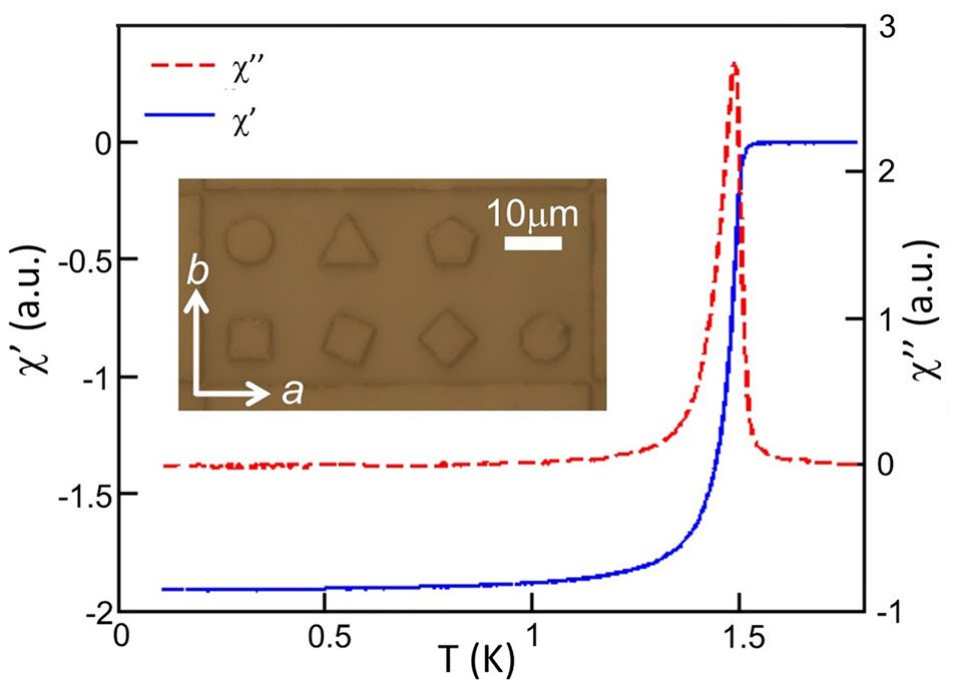
Real ($\chi^{\prime }$) and imaginary ($\chi^{\prime \prime }$) parts of the Sr_2_RuO_4_ single crystal ac susceptibility data measured through the critical temperature prior to annealing. An optical micrograph of the array of mesas etched into the *ab* face is shown in the inset.

Superconducting Sr_2_RuO_4_ single crystals were grown in a commercial image furnace using the floating-zone technique with a Ru self-flux^19^ and subsequently annealed in air at 1500 °C for 3 days to remove lattice defects and diminish vortex pinning^20^. The real ($\chi^{\prime }$) and imaginary ($\chi^{\prime \prime }$) components of ac susceptibility measurements (drive frequency 71 Hz, drive amplitude 0*.*43 G) of a single crystal sample before annealing and patterning are shown in Fig. 2. Defining T_c_ as the temperature where $\chi^{\prime }$ falls to 10% of its low temperature value and the transition width as its full width at half maximum we find T_c_ = 1.50 ± 0.03 K. This is extremely sharp for Sr_2_RuO_4_ indicating the very high crystalline quality of our crystals. Optical lithography with Shipley S1813 photoresist and Ar ion milling were used to etch multiple arrays of shallow pillars (depth ~ 400 nm) with regular geometrical shapes (triangles, squares, pentagons, octagons, circles) into the freshly cleaved *a-b* surface of a crystal. Selected facets of the mesa shapes were carefully aligned with the *a*/*b* lattice vectors of the underlying tetragonal crystal which had previously been established by x-ray diffraction (c.f., the optical micrograph inset of Fig. 2). The square mesas were designed to have side lengths of 10 μm, and the dimensions of the other shapes were chosen so that all had the same surface area of 100 μm^2^. The definition of the transferred pattern after etching was generally very good, in particular the squares show long straight edges with only slight rounding of the corners. Each sample has been patterning with over a hundred sets of mesas, each set containing one example of each mesa shape. We have investigated a number of different mesas and unpatterned regions across the sample. While there are small quantitative differences in, for example, the shape and positioning of vortex clusters as well as structures in the linescans across mesas, we saw the same qualitative behaviour everywhere that we looked.

Scanning Hall microscopy (SHM) has been used to map the stray magnetic fields just above the *a-b* surface of the sample. This technique uses piezoelectric transducers to approach and scan the sample with a nanoscale Hall effect sensor^21^. The active area of the sensor is defined by the intersection of two 800nm wide leads patterned in an AlGaAs/GaAs heterostructure using electron-beam lithography and wet chemical etching. The chip is equipped with an integrated scanning tunnelling microscopy (STM) tip formed by the evaporation of a thin film of gold over the corner of a deep mesa etch. During operation the plane of the sensor is tilted approximately 1° with respect to the sample surface so that the STM tip is always the closest point. It is approached towards the sample until a tunnelling current is established in a feedback loop and then manually retracted a few hundred nanometres from the surface to allow rapid imaging rates (~ 20 µm/s) without automated height control. In these experiments the sample-sensor separation is somewhat larger than we would typically use due to the strong surface topography of our patterned sample. By fitting profiles of individual vortices we estimate that the active Hall element sits ~ 1.23 µm above the sample surface during imaging due to a combination of the effects of ‘lift-off’ and tilt angle.

The scanner head was attached to the end of a Heliox VT-50 ^3^He refrigerator with the superconducting crystal mounted on a 12 mm diameter brass disk sample holder using Ag paint. A Cernox temperature sensor was embedded in the sample holder disk in addition to a RuO_2_ thermometer which was fixed to the Heliox ^3^He-pot. The sample holder was directly attached to the ^3^He-pot by means of a heavy copper braid to ensure good thermal anchoring and low temperature tests down to ~ 300 mK demonstrated that an almost perfect temperature equilibrium is established between the two thermometers with a time constant of about 1 s^21^.

## Results

All SHPM images were recorded just above the *a-b* surface of the crystal with the magnetic field applied along the *c* axis direction. In all cases the sample had been field-cooled in the stated magnetic field from just above T_c_ to the target temperature. In this way we have realised close to equilibrium vortex patterns, unperturbed by the strong screening effects that arise during zero field-cooling protocols.

**Figure 3:**
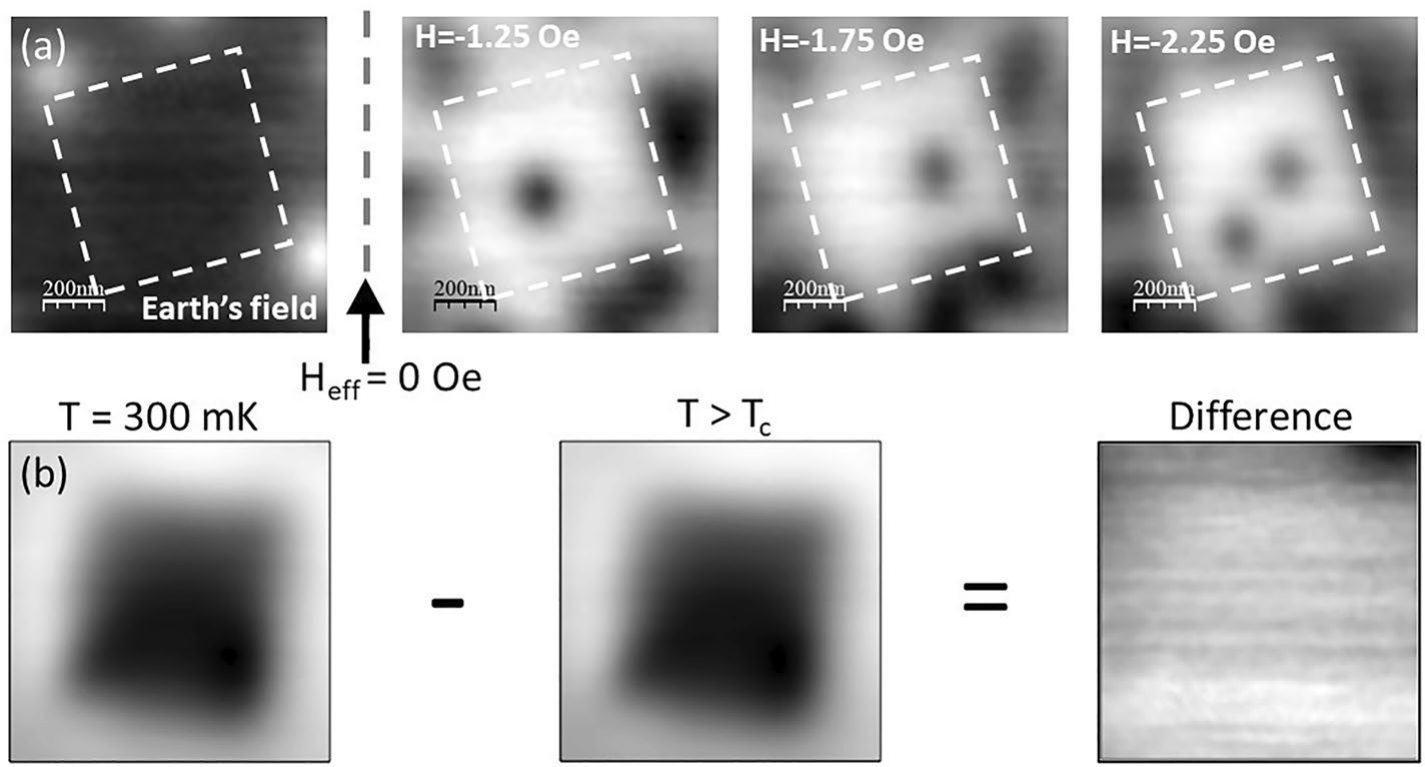
(**a**) Scanning Hall images (14 µm × 14 µm) of a 0° square Sr_2_RuO_4_ mesa after field-cooling to 300 mK in the indicated applied fields. (**b**) Illustration of the subtraction protocol used to remove image artefacts due to topographic ‘gating’.

In order to be able to distinguish possible spontaneous edge currents from conventional Meissner screening currents it is important to cancel out the earth’s field and any remnant field contributions from the cryostat as precisely as possible. Figure 3a shows a sequence of images that illustrates how this can be achieved with better than single vortex precision by capturing images over a narrow range of applied fields with the scanner centred over a square mesa. At very low effective magnetic fields vortices nucleate at preferential pinning sites just outside the square mesa whose outline is indicated by the white dashed line. We define H_eff_ = 0 as being at a point halfway between the field at which the first white vortex is nucleated and that at which the first black vortex is nucleated. Using this definition we estimate that H_earth_ = 0.43 ± 0.05 Oe, where the error bar is set by assuming we can at best detect vortices with a precision of a quarter of a flux quantum. Due to the statistical nature of vortex nucleation the uncertainty in H_eff_ = 0 does inevitably result in the occasional nucleation of a single vortex in a region that is intended to be flux free. Fortunately this can easily be distinguished from the field due to spontaneous edge currents on the basis of the expected symmetry. Figure 3a also shows that vortex nucleation in the square mesa itself only occurs at somewhat higher applied fields, i.e., H ~ 1.25 Oe for the first black vortex and H ~ 2.25 Oe for the second one.

**Figure 4:**
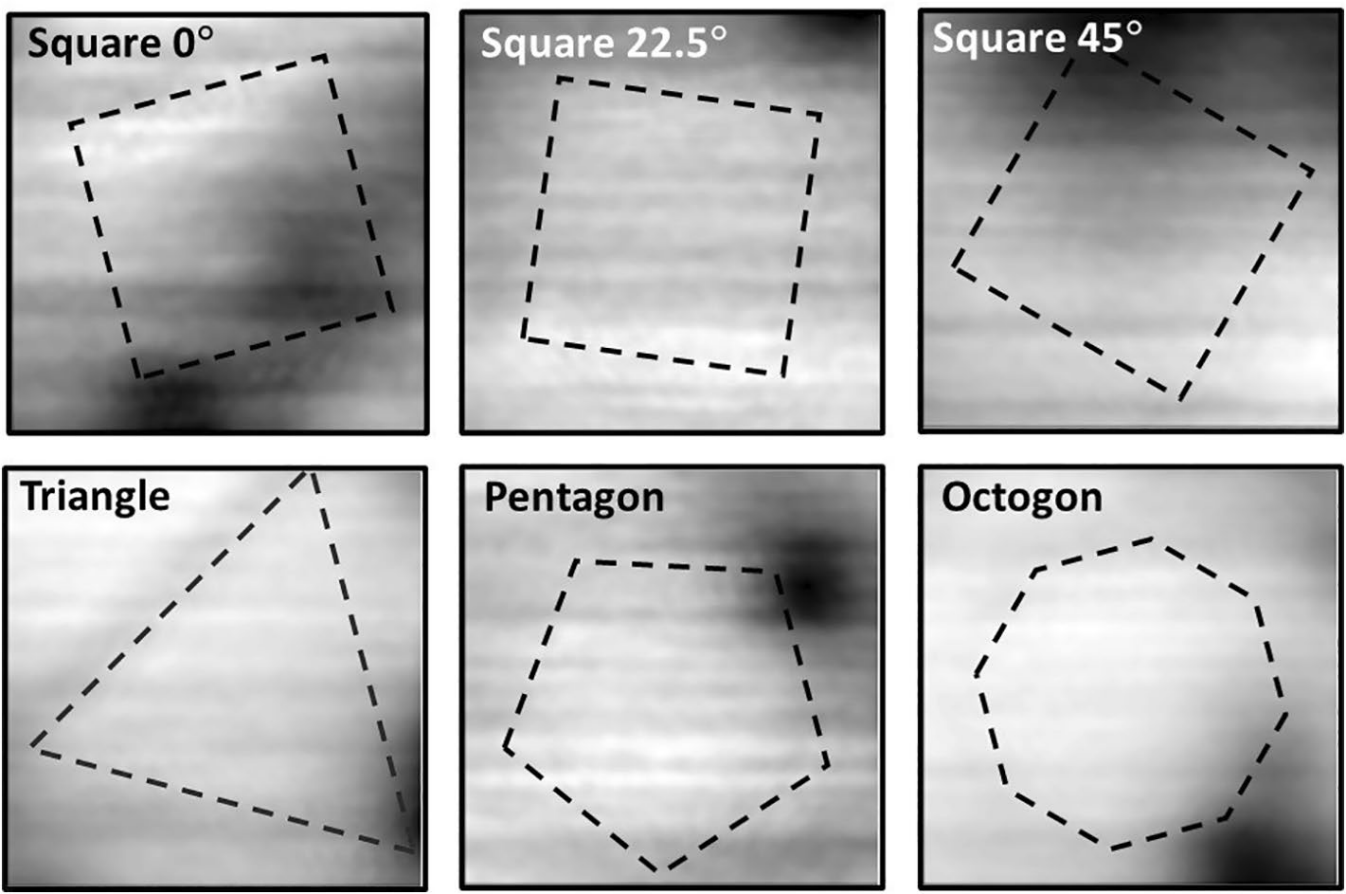
Compensated SHM images (14 µm × 14 µm) after field-cooling very close to H_eff_ = 0 for six mesa structures with different shapes and orientations. The superimposed dashed lines indicate the footprint of the patterned mesas.

**Figure 5:**
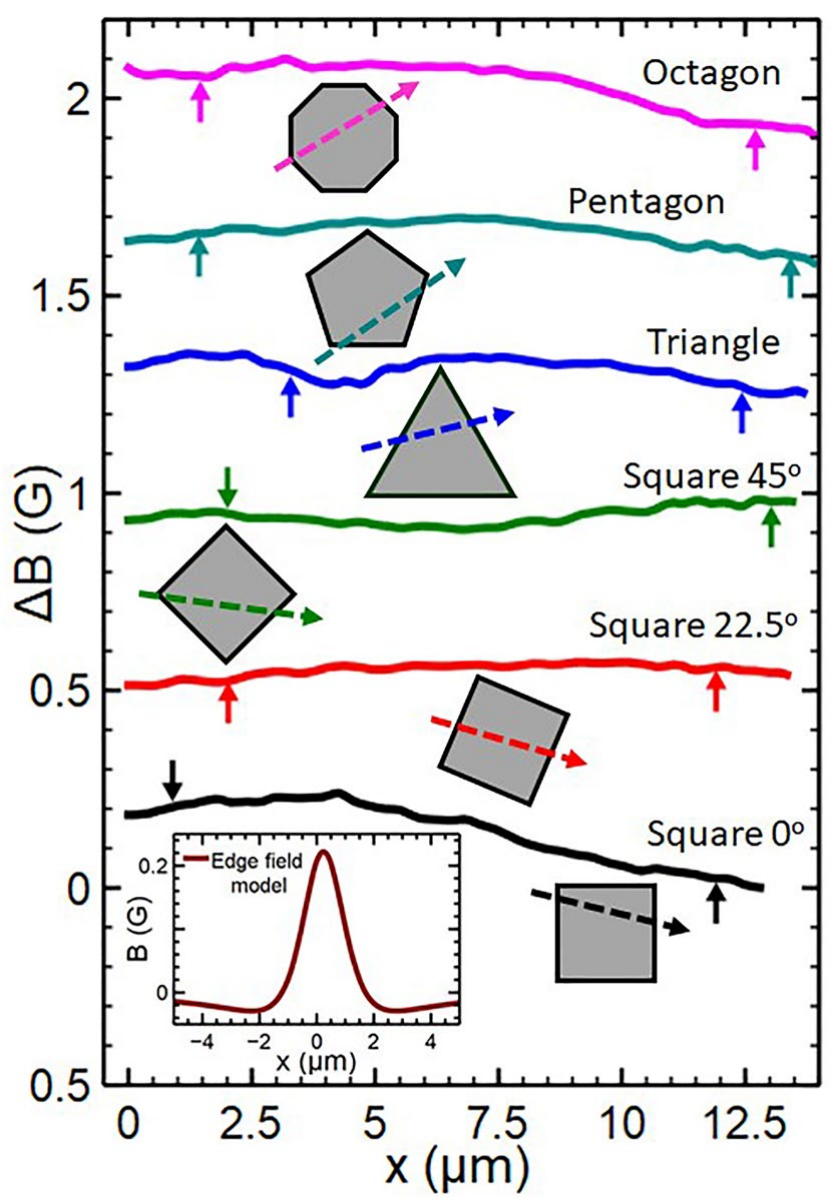
Linescans across the compensated SHM images of Fig. 4 in the indicated directions. The vertical arrows indicate the points where the linescan crosses the edges of the mesas. The inset shows a calculation of the expected edge field based on an approach described in reference^22^.

Even though samples are cooled at H_eff_ = 0, a region of weak image contrast is still observed over the mesa due to electrostatic ‘gating’ of the Hall sensor by the sample which is at a relative bias of + 0.2 V. This potential difference is essential in order to allow surface detection via tunnel currents from the grounded STM tip, but results in an imaging artefact because the strong sample topography leads to a spatially dependent electric field. However, since the gating signal is independent of temperature it can be almost completely removed by subtracting an image taken just above T_c_ from the image of interest below T_c_, provided the field of view is adjusted by a small amount (~ 1–2%) to account for the temperature dependence of the piezotube. Figure 3b illustrates how this is achieved by subtraction of the image of a square mesa captured at 1.6 K (T > T_c_) from an image at 0.3 K at H_eff_ = 0. This shows that the black gating artefact above the mesa is very effectively removed by constructing the difference image. Indeed, apart from the partial black vortex in the top right hand corner there appears to be no magnetic contrast in this image above the noise level of our measurement of approximately ± 0.025 G. Figure 4 shows difference images produced using this procedure for three square mesas with different orientations, a triangle, a pentagon and an octagon. Although a partial black vortex appears in several images, we find no credible evidence for additional fields due to spontaneous edge currents in any of the mesas and nothing corresponding to our expectations from Fig. 1. Any residual dark contrast is almost certainly due to imperfect subtraction of the normal state reference image. Figure 5 plots linescans across the images of Fig. 4 along the indicated directions. For comparison, in the inset we also show a calculation of the expected edge field profile for an infinite straight mesa edge following the fitting approach of Bluhm^22^ to approximate numerical solutions of the inhomogeneous London equation for spontaneous currents at the edge of a single domain sample given by Matsumoto and Sigrist^23^. We have used the same fit parameters (λ = 150 nm, ξ = 66 nm, $\widetilde{\uplambda }$ = 2.2ξ, $\widetilde{\upxi }$ = 1.5ξ and B_0_ = 87 G) assumed by Bluhm, an active Hall probe width of 0.5 μm and a scan height of 1.23 μm. This is plotted in the lower right inset of Fig. 5 and shows that we expect these fields to be peaked just inside of the mesa with a magnitude up to ~ 0.25 G and a full width at half maximum of ~ 1.5 µm. Moreover, the fields should reverse sign as one traverses around the perimeter of the triangle, pentagon and octagon. Although the traces in Fig. 5 are not completely featureless due to imperfect background subtraction, none of them show structures consistent with the presence of spontaneous edge currents above our ± 0.025 G noise floor.

**Figure 6:**
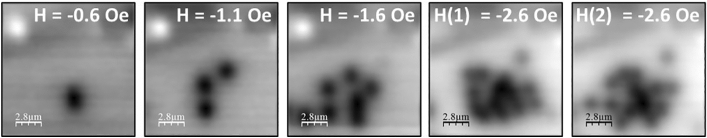
SHM images (14 µm × 14 µm) captured after field-cooling to 300 mK in the indicated applied field in an unpatterned region of the sample.

**Figure 7:**
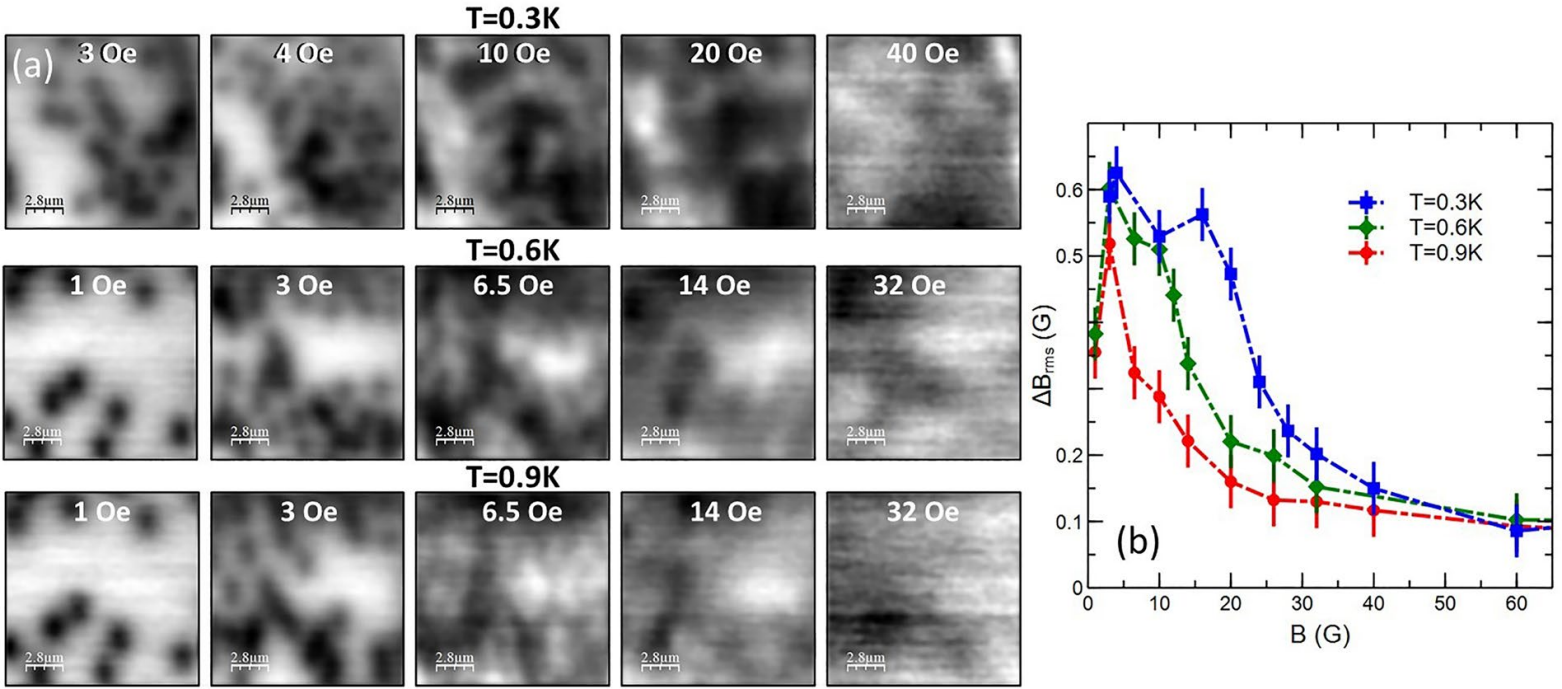
(**a**) SHM images (14 µm × 14 µm) captured in an unpatterned region of the sample after field-cooling to 0.3 K (top row), 0.6 K (middle row) and 0.9 K (bottom row) in the indicated applied magnetic fields. (**b**) RMS magnetic field ‘roughness’ as a function of cooling field and measurement temperature.

It is well documented that novel flux structures can nucleate in low kappa (κ = λ/ξ) materials with values of the Ginzburg–Landau parameter close to 1/√2. In the so-called type II/1 regime the vortex interaction acquires an attractive component leading to an intermediate mixed state (IMS) composed of vortex clusters in a vortex-free “matrix”^24–27^. κ ~ 2.3 in Sr_2_RuO_4_ in our measurement geometry which is small enough that some IMS physics might still possibly be observed. Previous scanning Hall^28^ and scanning SQUID^14,16,28^ microscopy images of Sr_2_RuO_4_ single crystals have been interpreted in terms of vortex coalescence due to a weak long-range vortex attraction at low fields. Strong evidence for this was also seen in muon spin rotation measurements as a function of the cooling field^29^ which was explained in terms of a semi-Meissner state that is predicted to arise in Sr_2_RuO_4_ from an interplay between the two orbital degrees of freedom of a chiral superconductor as well as its multiband nature^30^. We have revisited this issue by measuring a series of images of vortex patterns in a flat region of the sample without any etched mesas. Figure 6 shows a set of images captured in the same position after field-cooling to 0.3 K at small fields in the range − 0.6 Oe to − 2.6 Oe. Vortex clustering is clearly observed, characteristic of a semi-Meissner system with multiple effective coherence lengths leading to long-range attractive and short-range repulsive forces between vortices. Note that the final two images have both been field-cooled at different times in H = − 2.6 Oe and show significantly different vortex patterns, confirming that the clustering is not simply driven by a large number of particularly strong pinning sites arising from quenched disorder in the sample. However, we do find that the clusters tend to nucleate in the same regions of the sample after each cooldown suggesting that sample disorder is playing some role. The most likely scenario is that the first vortex nucleates at a particularly strong pinning site in the sample and the cluster then forms around this driven by the mutual vortex-vortex attraction. Figure 7a shows the results of a systematic study of the temperature-dependence of this effect up to much larger applied magnetic fields. Figure 7b plots the rms ‘roughness’ of magnetic field maps as a function of applied field at three different temperatures. For all but the very lowest fields where only few vortices are present, a high value of ΔB_rms_ indicates strong vortex clustering, and we see that this becomes abruptly suppressed above a characteristic temperature-dependent applied field. If we focus on the points where ΔB_rms_(B) has its steepest slope, this behaviour is qualitatively very similar to the field-dependence of the flux containing fraction of the sample inferred from muon spin rotation measurements in reference^29^. If we compare the values of field and temperature where ΔB_rms_ falls to 50% of its peak value (~ 24 G at 0.3 K, ~ 15 G at 0.6 K and ~ 10 G at 0.9 K) with the points where the fraction of vortex containing regions in Fig. 3 of reference^29^ is 50% these exhibit a very similar trend, although our threshold SHM field values are substantially lower than µSR ones by a factor of approximately three.

**Figure 8:**
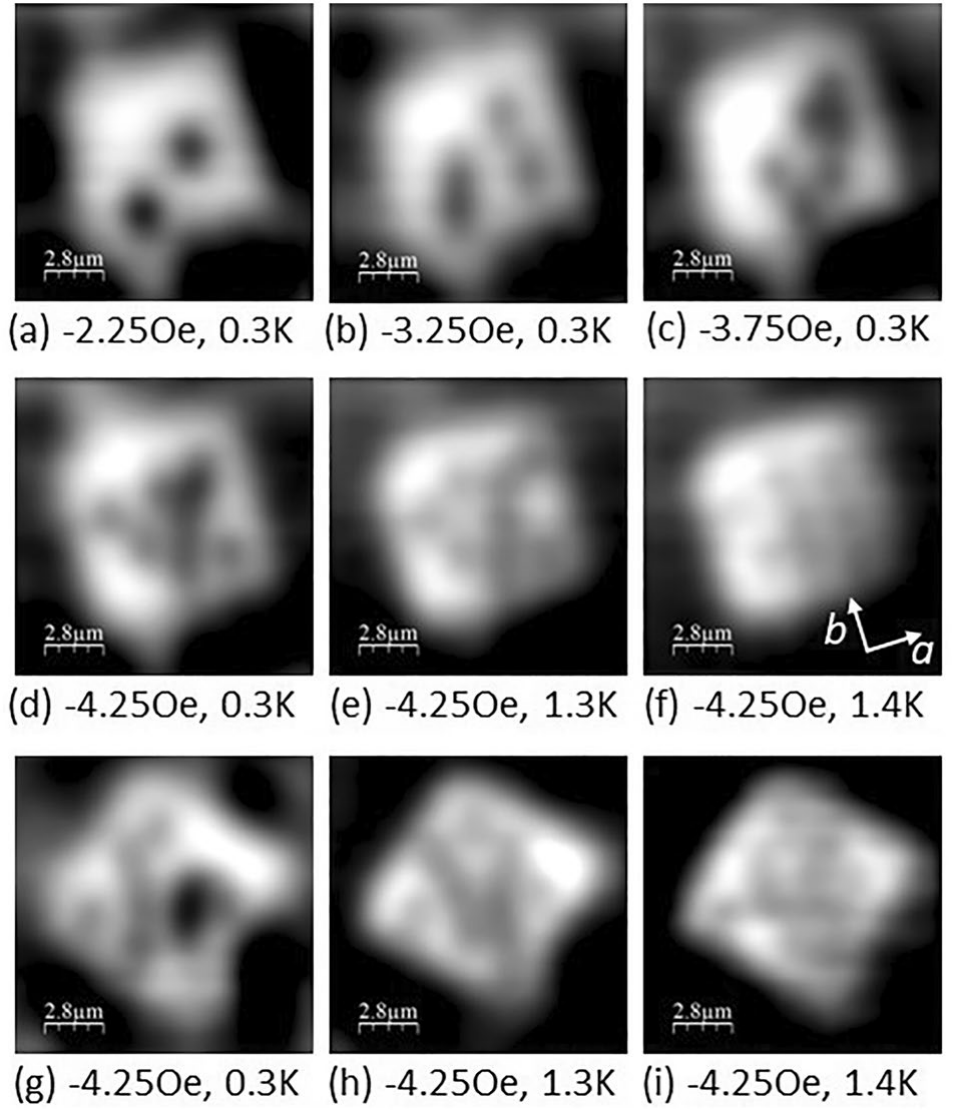
SHM images (14 µm × 14 µm) of (**a**)–(**f**) a 0° square mesa and (**g**)–(**i**) a 45° square mesa (bottom row) after field cooling in the various listed fields to the indicated temperatures.

The tendency for clustering is also clearly observed in vortex structures forming inside the mesa structures themselves as illustrated in Fig. 8. Images Fig. 8a–c show vortices in the 0° square mesa after field-cooling to 0.3 K in increasingly higher applied fields. After the first two vortices nucleate at − 2.25 Oe, it is clear that the distributions become strongly bunched, particularly at − 3.75 Oe. Figure 8d–f show the same mesa structure after field-cooling to 0.3 K in − 4.25 Oe as the temperature is raised to 1.3 K and 1.4 K. The low temperature state is highly clustered, but the vortex distribution becomes much more dilute at 1.3 K, with clear evidence for a quite well-ordered square vortex lattice structure at 1.4 K with one of the lattice vectors parallel to the underlying crystalline b-axis. Figure 8g–i show a similar study of the temperature dependence of the 45° square mesa after field-cooling to 0.3 K at − 4.25 Oe. Again the low temperature clustered state becomes much more ordered at high temperatures with a predominant vortex chain direction parallel to the crystalline b-axis. However, there is little evidence for a square vortex lattice in the 1.4 K image (Fig. 8i), probably because it has been destabilised by the interaction with screening currents flowing at the edges of the mesa which in this case are not parallel to the vortex lattice vectors.

**Figure 9:**
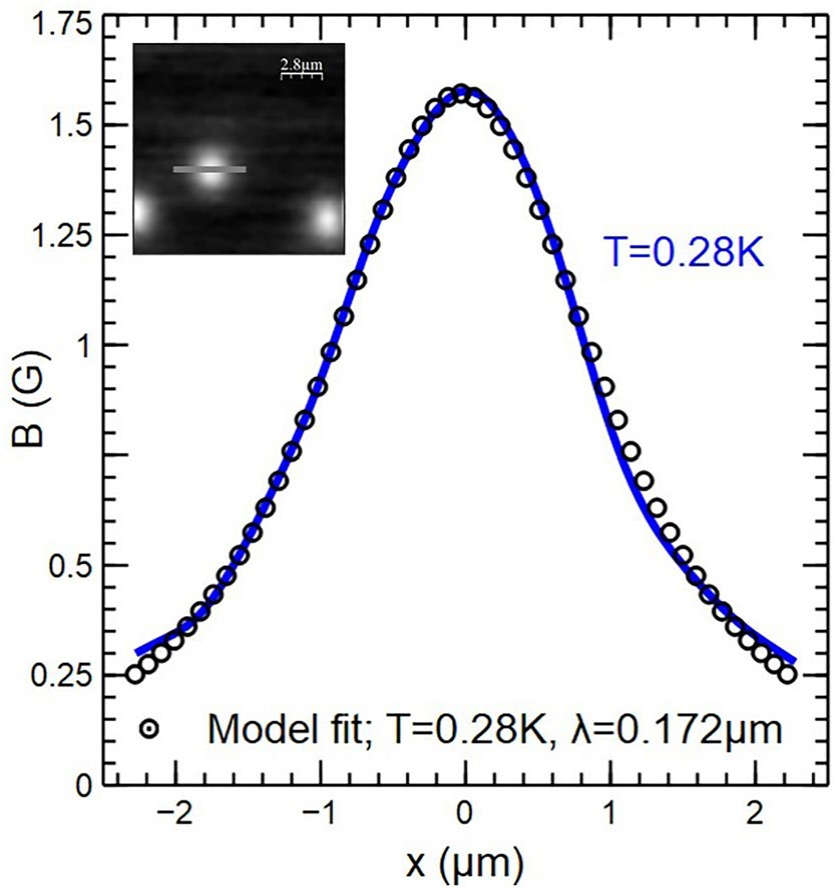
Measured vortex profile at T = 0.28 K (solid line) and a fit to a modified Clem model (circles). The profile has been measured along the line indicated in the T = 0.28 K SHM image (14 µm × 14 µm) shown in the inset.

Finally we have fitted the profile of a well-isolated vortex in an unpatterned region of the sample at very low applied magnetic field. Figure 9 shows a profile across the vortex measured at T = 0.28 K after field-cooling in *H* = 0*.8* Oe. Also shown is a fit to the T = 0.28 K linescan based on the Clem variational model^31^ modified to account for surface screening effects using an approach due to Kirtley et al.^15^ and assuming a variational coherence length ξ_v_(0) = 66 nm, λ(0) = 167 nm, and an active Hall probe width, w, of 500 nm. In practice the active electronic Hall probe width is reduced from its geometrical width of 800 nm due to sidewall depletion of carriers at the etched surfaces.

1 $$\begin{gathered} {\text{B}}\left( {{\text{x}},{\text{y}},{\text{z}}} \right) = \frac{{{\Phi }_{0} }}{{{\text{w}}^{2} }}\mathop \int \limits_{{{\text{y}} - \frac{{\text{w}}}{2}}}^{{{\text{y}} + \frac{{\text{w}}}{2}}} \mathop \int \limits_{{{\text{x}} - \frac{{\text{w}}}{2}}}^{{{\text{x}} + \frac{{\text{w}}}{2}}} \mathop \int \limits_{0}^{\infty } \frac{{{\text{K}}_{1} \left( {\sqrt {{\text{k}}^{2} + \lambda^{ - 2} } \xi_{{\text{v}}} } \right)}}{{2\pi \lambda \left( {\sqrt {{\text{k}}^{2} + \lambda^{ - 2} } + {\text{k}}} \right){\text{K}}_{1} \left( {\xi_{{\text{v}}} /\lambda } \right)}} \hfill \\ \quad \quad \quad \quad \quad \times \;{\text{J}}_{0} \left( {{\text{k}}\sqrt {{\text{x}}^{\prime 2} + {\text{y}}^{\prime 2} } } \right){\text{exp}}\left( { - {\text{kz}}} \right){\text{kdkdx}}^{\prime } {\text{dy}}^{\prime } \hfill \\ \end{gathered}$$

where *z* is the scan height of the sensor above the surface of the superconductor which has been treated here as a fit parameter and found to be 1.23 ± 0.01 μm. The modified Clem model appears to provide a rather good description of the measured field profiles, although the fitted scan height is significantly larger than one would expect based on the sensor geometry (typically 0.5 µm for a 1° tilt angle). This parameter is normally set by the spatial separation between the STM tip and the active Hall cross on the sensor chip, and the tilt angle between the sensor plane and the sample surface, suggesting an unfeasibly large tilt angle. A similar level of unexplained vortex broadening has been observed in previous SHM measurements^32^ and could possibly be related to some form of surface scattering or motional broadening due to vortex fluctuations about their pinning sites. Equation (1) assumes that vortices all contain a single flux quantum and our quantitative scanning Hall measurements allow us to test this assumption. Although there are some numerical issues associated with integrating the flux when two or more vortices are close together, we have satisfied ourselves that all images are fully consistent with single quantum vortices.

## Discussion

As was also observed in our previous work on mesoscopic disks^17^, etching mesas into the surface of a high quality Sr_2_RuO_4_ single crystal has a profound impact on the nucleating vortex structures. At very low fields (c.f., Fig. 3a) strong edge currents screen the mesas and prevent vortex penetration into them, forcing vortices to occupy pinning sites just outside the mesa edges. Upon penetration at higher fields the first vortex moves towards the mesa centre driven by screening currents flowing around the perimeter, but its exact location is strongly influenced by interactions with vortices pinned just outside the mesa. The statistical nature of vortex nucleation limits our ability to fine tune the applied field to realise perfect ‘null’ images that are entirely free of vortices. However, the local nature of vortex stray fields makes it trivial to distinguish them from fields arising due to spontaneous edge currents. The analysis is further complicated by the need to compensate images for gating effects arising from the very strong surface topography. Nevertheless we find no credible evidence for spontaneous currents near the mesa edges (or due to chiral domain walls) that could be attributed to a chiral order parameter within the ± 25 mG experimental noise floor of this experiment. This is approximately an order of magnitude smaller than estimates of the edge field based on the approach of Bluhm^22^ (c.f., inset of Fig. 5) using his parameter set which was estimated from fits to the numerical results of reference^23^.

Imaging experiments in unpatterning regions of the single crystal sample clearly show evidence for a long range attractive component to the vortex-vortex interaction that leads to vortex clustering. Moreover, we find that qualitatively different vortex cluster patterns form after different cooldown cycles at the same magnetic field, indicating that this is not simply related to the presence of a few very strong pinning sites in the sample. The high field behaviour as a function of temperature shown in Fig. 7 is very strongly reminiscent of previous μSR experiments which observed a dramatic increase in the vortex-free Meissner fraction at low temperatures due to pronounced vortex clustering, something that was tentatively attributed to a long-range vortex attraction arising from multiband effects^29^. We note that the locus of field/temperature points where ΔB_rms_ falls to 50% of its peak value in Fig. 7b is qualitatively very similar to the 50% vortex containing fraction line in Fig. 3 of reference^29^. The approximate factor of 3 difference in the measured threshold fields is likely to be a consequence of the very different types of experiments used. The µSR experiment is a bulk probe and data have been ensemble averaged over a mosaic of many single crystals, while SHM is a surface probe that only captures data from a very small ~ 14 µm × 14 µm region near the centre of the sample. Similar vortex clustering effects are also observed inside mesa structures at low temperatures as shown in Fig. 8 for 0° and 45° square geometries. As in unpatterned regions, the clustering is strongly suppressed at higher temperatures (c.f., images at 1.3 K and 1.4 K) when vortices spread out to occupy most of the mesa footprint. For the 0° square there also is a clear tendency for vortices to order into a square lattice at 1.4 K with one of the lattice vectors directed along the crystallographic b-axis, something that is probably reinforced by interactions with screening currents flowing around the perimeter of the mesa. The 45° square sample also seems to show pronounced vortex chains forming close to the b-axis direction at high temperatures, but in this case, there is no clear evidence for square lattice ordering, probably because interactions with screening currents at the perimeter tend to destabilise it in this geometry. These observations are fully consistent with the results of previous SHM studies of vortices in an unpatterned Sr_2_RuO_4_ single crystal where a field- and temperature-driven structural transition was observed from a triangular lattice at low field and temperature to a square lattice at high field and temperature^32^. This was interpreted in terms of a model developed by Heeb and Agterberg^33^ who investigated the ground state vortex structure in Sr_2_RuO_4_ as a function of Fermi surface anisotropy and applied field using extended London theory for a two component *p*-wave order parameter. They predicted a continuous triangular → rectangular → square field-driven transition, consistent with our observations.

## Conclusions

Geometrically shaped mesas, whose sides are carefully aligned with respect to the underlying crystallographic axes, have been milled into the *ab* surface of high quality Sr_2_RuO_4_ single crystals in order to test recent theories of the direction of spontaneous edge current flow as a function of facet orientation and band filling^18^. Independent of the shape or orientation of mesas, scanning Hall imaging reveals no credible evidence for spontaneous magnetic signals at the edges of these structures above the experimental noise threshold of ± 25 mG, placing an upper limit of ≈ 10% of theoretical predictions. In addition, we do not observe magnetic signals elsewhere in the sample that could be attributed to spontaneous currents at chiral domain walls. Measurements in unpatterned regions of the sample reveal strong evidence for vortex clustering at low applied magnetic field and temperature indicating a long range attractive component to the vortex-vortex interaction. As the field is increased, we see a reasonably abrupt temperature-dependent transition to much more homogenous vortex patterns. This behaviour is consistent with the established semi-Meissner scenario whereby the nucleation of vortex clusters arises from the multiband nature of the superconductivity. In a regime with multiple coherence lengths arising from active and passive bands, calculations^30^ have shown that an attractive intervortex interaction can appear below T_c_ leading to an energetically preferred intervortex distance that is considerably smaller than the equilibrium separation which would be expected for strictly repulsive interactions. Vortex clustering is also clearly visible at low temperatures within the patterned mesas themselves, with evidence for the formation of a square vortex lattice at higher temperatures, with one of the lattice vectors directed along the crystalline b-axis. While the apparent absence of spontaneous edge currents tends to confirm the current view that Sr_2_RuO_4_ is not a chiral p-wave superconductor, the observation of vortex clustering at low fields and temperatures as well as the transition to a square vortex lattice represent both an impetus and a challenge for the development of realistic models for superconductivity in this remarkable material.

## Data Availability

The data that support the findings of this study are openly available in the University of Bath Research Data Archive at https://doi.org/10.15125/BATH-01304.
